# Pediatric Horner’s syndrome following posterior spinal fusion: a case series

**DOI:** 10.1007/s43390-026-01305-1

**Published:** 2026-02-19

**Authors:** Judy-Mae Lima, Amy L. McIntosh, Daniel J. Sucato, Megan E. Johnson

**Affiliations:** 1 Department of Orthopedic Surgery, Texas Scottish Rite Hospital for Children, Dallas, TX USA; 2 Department of Orthopedic Surgery, UT Southwestern, Dallas, TX USA

**Keywords:** Horner’s syndrome, Pediatric scoliosis, Post-operative complication

## Abstract

**Purpose:**

This case series aims to highlight Horner’s syndrome, a clinical diagnosis of unilateral ptosis, miosis, and anhidrosis, as a rare complication of posterior spinal surgery in pediatric patients.

**Methods:**

In this series, we present three pediatric patients who developed Horner’s syndrome after surgical correction of scoliosis involving the cervicothoracic region.

**Results:**

Case 1 is a 12-year-old female with DiGeorge syndrome who underwent revision surgery with extension into the cervical spine after developing proximal junctional kyphosis 2 years after her index spinal fusion procedure. Case 2 is a 9-year-old girl who had posterior spinal fusion for progressive thoracic congenital scoliosis. Case 3 is a 4-year-old female with Klippel–Feil syndrome who underwent correction of a cervicothoracic curve and hemivertebrae excision. Postoperatively, all three patients were noted to have either unilateral miosis or ptosis, with an eventual diagnosis of acquired Horner’s syndrome.

**Conclusions:**

This case series demonstrates that Horner’s syndrome is a rare complication after a posterior spinal fusion in the pediatric population. Each case highlights that the oculosympathetic pathway can be susceptible to injury during deformity correction, specifically near the cervicothoracic region, whether from traction injury or overt nerve root transection.

## Introduction

Horner’s syndrome is a clinical diagnosis characterized by the classic triad of unilateral ptosis (drooping of the upper eyelid), miosis (pupillary constriction), and anhidrosis (decreased sweating) on the affected side of the face. These signs result from disruption of the oculosympathetic pathway, a three-neuron chain connecting the hypothalamus to the ipsilateral eye. Lesions at any point along this chain can produce Horner’s syndrome [1].

First-order neuron lesions originate between the hypothalamus and the lower cervical/upper thoracic spinal cord from central causes, such as tumors, hemorrhage, or demyelinating disease. Second-order/preganglionic lesions occur between the spinal cord (C8 to T2) and the superior cervical ganglion and are often associated with upper thoracic region pathologies, such as Pancoast tumors, brachial plexus injuries, or surgical intervention/trauma. Third-order/postganglionic lesions involve the pathway from the superior cervical ganglion along the carotid artery system to the ophthalmic branch of the trigeminal nerve, with causes, such as carotid dissection [1].

Iatrogenic Horner’s syndrome following spinal surgery is rare, particularly in pediatric patients. One study found this complication present in 0.06% of anterior cervical spinal surgeries and an even smaller number have been reported after posterior approaches [2]. Isolated pediatric case reports have described Horner’s syndrome as a complication in varying orthopedic spine surgeries, including posterior vertebral column resection for congenital kyphoscoliosis and posterior spinal surgery for idiopathic syringomyelia, as examples [3, 4]. To our knowledge, this is the largest pediatric case of Horner’s syndrome following posterior spinal fusion (PSF) at a single-site pediatric orthopedic hospital—one patient with DiGeorge syndrome and two with congenital scoliosis, as summarized in Table 1.

**Table 1:** Summary of three pediatric patients identified at a single pediatric orthopedic hospital who obtained Horner’s Syndrome following posterior spinal surgery to address scoliosis

Case number	Age	Sex	Scoliosis diagnosis	Underlying diagnosis	Procedure complete	Spinal levels instrumented	Use of traction	Intra-operative events	Onset on Horner’s sign	Diagnostic confirmation method	Treatment	Duration
1	12	Female	Neuromuscular	DiGeorge syndrome	Revision instrumented posterior spinal fusion C5-L2; Ponte osteotomy T1-T2	C5-L2	Halo Gravity preoperatively, intraoperative head and feet	Neuromonitoring changes during C5 and C6 instrumentation	POD 1	Clinical diagnosis of ptosis, miosis, facial swelling, and upper extremity weakness	Gabapentin	15 months
2	9	Female	Congenital	None	Posterior spinal fusion T1–T9; vertebral column resection T4–T6, bilateral Rib resction	T1-T9	No	None	POD 0	Clinical diagnosis of ptosis, facial edema, and upper extremity weakness	None	4 years
3	4	Female	Congenital	Klippel-Feil syndrome	Posterior spinal fusion C4–T5; Left T2 hemivertebectomy	C4–T5	No	T2 nerve root transection	POD 0	Clinical diagnosis of ptosis, miosis, and upper extremity pain	Observation	7 years and 7 months

## Case reports

### Case 1

A 12-year-old female with DiGeorge syndrome presented for evaluation of worsening head and neck posture following prior posterior spinal fusion T2–L1 for syndromic scoliosis. Syndromic scoliosis was diagnosed at age 2 and monitored before bracing was initiated. At age 9, she underwent posterior spinal fusion and instrumentation from T2 to L1 for a 60-degree right thoracic curve. Three years later, the patient developed proximal junctional kyphosis (PJK) above the construct at T1–T2, initially managed by physical therapy. Progressive radiographic and clinical worsening prompted revision posterior spinal surgery.

**Fig. 1:**
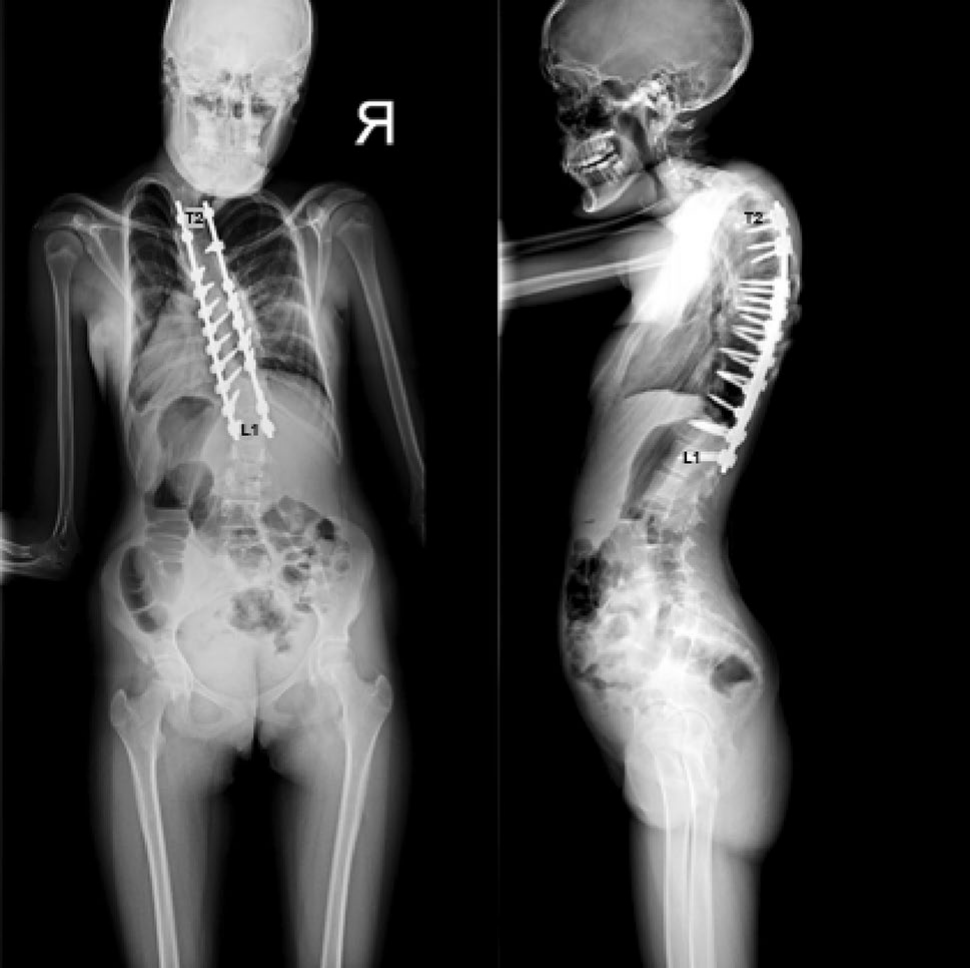
Case 1: Preoperative PA and lateral X-ray of a 12-year-old female with DiGeorge syndrome and syndromic scoliosis

Pre-operative examination demonstrated the patient to be at her neurological baseline. MRI and CT imaging revealed that the previous hardware to be intact from T2 to L1, with PJK proximal to the construct. A trunk shift to the left and a compensatory head tilt to the right were noted. Radiographs showed 57-degree T1–T12 thoracic kyphosis as well as 72-degree lumbar lordosis (Fig. 1).

**Fig. 2:**
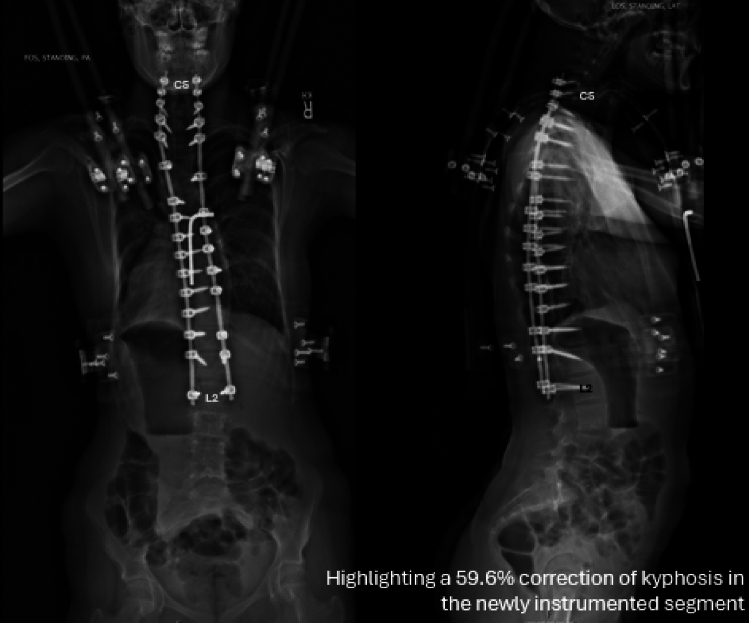
Case 1: Postoperative PA and lateral X-ray of a 12-year-old female with DiGeorge syndrome and syndromic scoliosis

The patient underwent 7 weeks of halo gravity traction (HGT) preoperatively followed by revision posterior spinal fusion, with extension of the original construct from C5 to L2. Pedicle screws were placed at C7 and T1 and lateral mass screws were placed at C5–C6 using 7D image navigation based on a preoperative CT. Surgical correction technique included in situ cantilever contouring for kyphosis correction, compression/distraction techniques, and a posterior column osteotomy at T1–T2, resulting in a 59.6% correction of kyphosis from 57° to 23°. Neuromonitoring changes occurred during screw placement at C5 and C6, including decreased motor evoked potentials (MEPs) in the right abductor pollicis brevis and abductor digiti minimi, as well as decreased somatosensory evoked potentials (SSEPs) in the ulnar nerve. We suspect that the neuromonitoring change was due to stretch during the PJK correction. These changes persisted despite intraoperative interventions including optimizing anesthesia and decreasing traction but returned to baseline by the time of closure (Fig. 2).

**Fig. 3:**
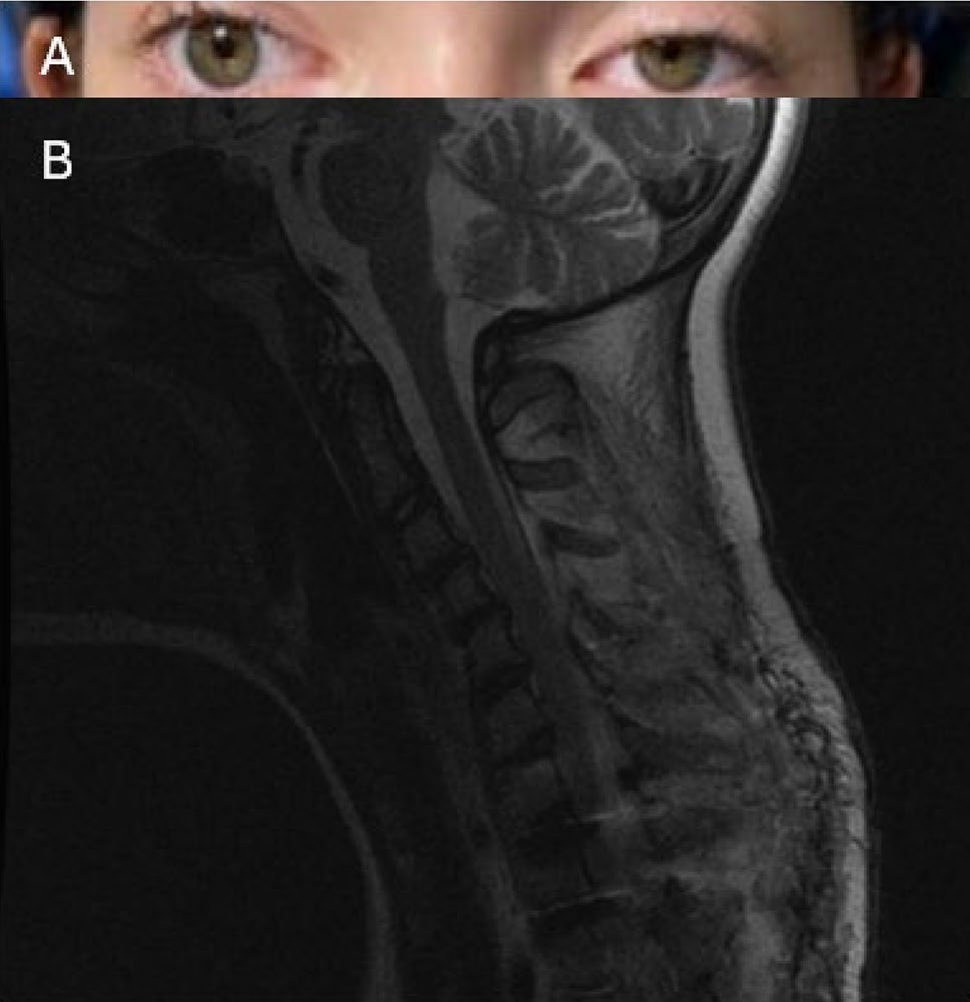
(**A**) Case 1 Clinical manifestation of ptosis and miosis; (**B**) MRI cervical spine lateral of a 12-year-old female with DiGeorge syndrome and syndromic scoliosis

Postoperatively, the patient was placed into a halo vest. On postoperative day (POD)1, her mother noted left eye swelling. She also developed left upper extremity pain (primarily the forearm), difficulty gripping objects, and dysphagia. By POD3, there was a notable pupillary difference with right greater than left and increased burping; however, anhidrosis was not assessed or noted at the time (Fig. 3A). All other neurologic exams remained at baseline with no other complications. She was discharged, and her symptoms were evaluated in the outpatient setting.

At a neurology appointment 1 week later, the exam was notable for left ptosis, miosis, facial swelling, and upper extremity weakness of 4+ out of 5 across all myotomes of the left upper extremity, consistent with Horner’s syndrome. However, no pharmacologic testing or pupillometry was performed to confirm the diagnosis. MRI of the cervical spine was limited due to the implant artifact, but showed no cord or nerve root impingement, or signal changes and hardware was appropriately positioned (Fig. 3B). Gabapentin 300 mg twice daily was prescribed for the patient’s neuropathic pain.

At 4 months postoperatively, left ptosis persisted with mild left cheek swelling and occasional burping/coughing/gagging without interfering with eating or drinking. Pain and left arm weakness had resolved, and at 15 months, her symptoms had nearly resolved with mild residual miosis, and Gabapentin was successfully weaned off. At 2-year follow-up, pupils were equal in size without pharmacologic intervention, ptosis had resolved, and neurologic exam showed no persistent functional impairment.

### Case 2

**Fig. 4:**
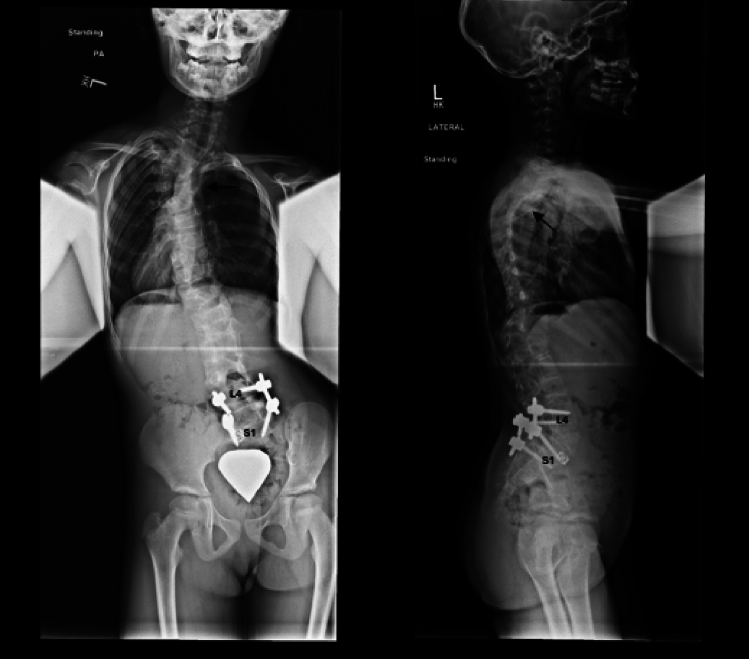
Case 2 Pre-operative PA and lateral X-ray of a 9-year-old female with congenital scoliosis

A 9-year-old girl with congenital scoliosis presented for surgical management of progressive deformity. Congenital scoliosis was first diagnosed at 3 months after presenting with torticollis. Radiographs revealed congenital anomalies of the spine including left-sided scoliosis with right-sided fused ribs, fused vertebrae, hemivertebrae at the lumbosacral junction, and spina bifida occulta (Fig. 4). Her prior treatments included physical therapy for the torticollis, tethered cord release at age 1, posterior spinal fusion from L4 to S1 with resection of L5 hemivertebra at age 5, and an attempted spinal fusion of T1–T9 at age 8, which was aborted due to spinal cord monitoring changes to the right lower extremity and transient postoperative weakness.

MRI obtained postoperatively demonstrated no spinal cord injury. Neurology consultation showed a return to previous neurological baseline. Therefore, surgical intervention was attempted again, including a PSF of T1–T9, vertebral column resection T4–T6, and a bilateral rib resection to address scoliosis as well as truncal shift at 9 years.

**Fig. 5:**
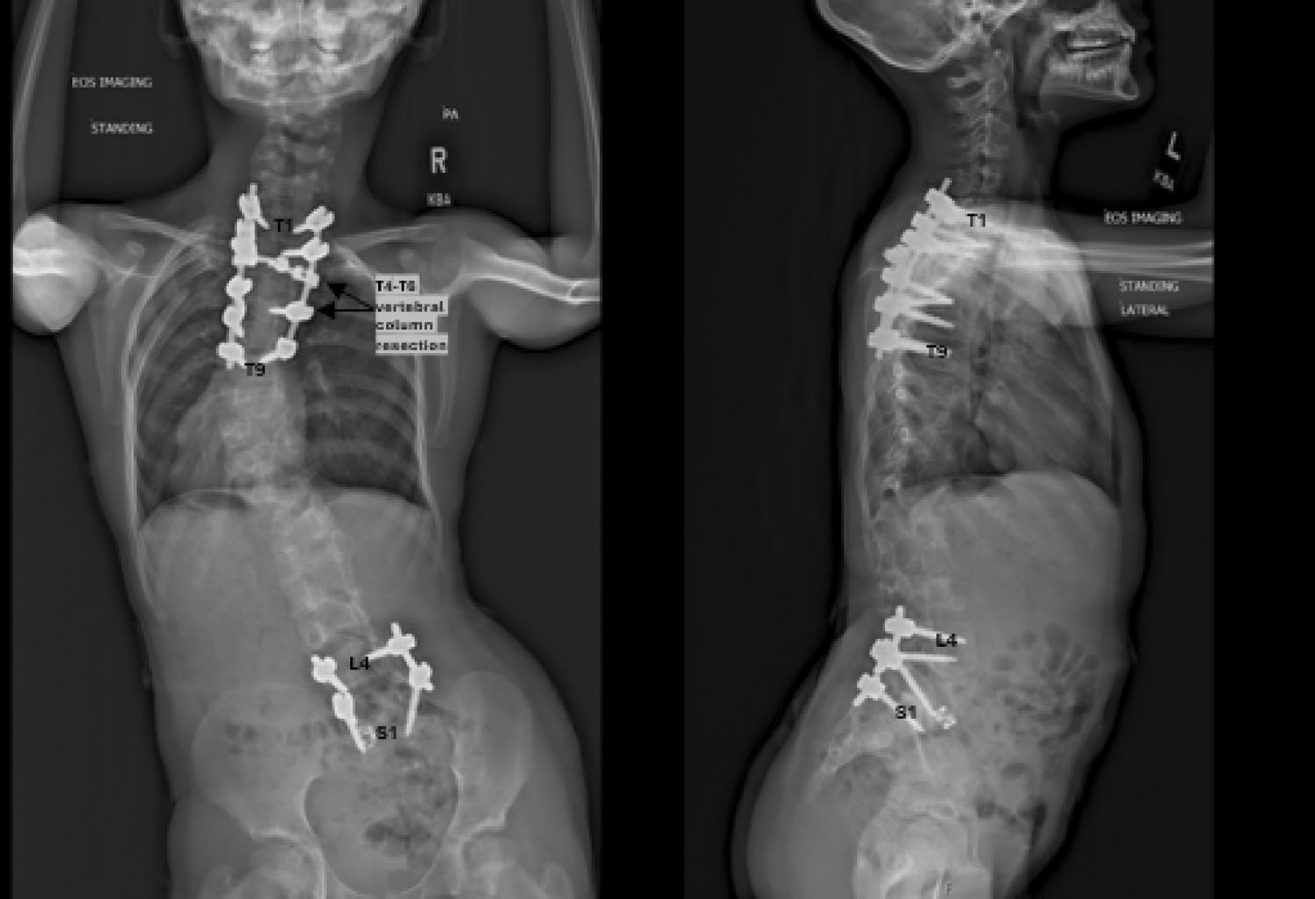
Case 2 Post-operative PA and lateral X-ray of a 9-year-old female with congenital scoliosis

The patient underwent general anesthesia with intraoperative SSEP and MEP monitoring. O-arm guidance was used for pedicle screw placement and stabilization from T1–T9. A posterior wedge-shaped vertebral column resection was used for the congenital fusion mass at T4–T6. Surgical correction technique for the deformity included cantilever reduction to reduce the vertebral column resection and compression on all screws. There were no intraoperative neuromonitoring changes (Fig. 5).

Postoperatively, the patient woke up neurologically intact and recovered uneventfully from surgery. At the 6-week follow-up, her mother reported a persistent left eye ptosis since the surgery. Initially attributed to facial edema, it persisted after discharge and was accompanied by numbness and weakness in the left upper extremity. Physical exam showed left ptosis, miosis, decreased sensation over the medial forearm and digits two through four, and 4 out of 5 strength in finger flexion, abduction, and grip strength. No anhidrosis was detected, and proximal upper extremity strength was preserved. Findings were consistent with Horner’s syndrome and lower trunk brachial plexopathy; however, no pharmacologic testing or pupillometry was performed to confirm the diagnosis. The decision was made to observe for symptom progression.

At 6 months postoperatively, symptoms showed gradual improvement. CT imaging showed adequately placed screws and intact posterior osseous fusion. At age 12, 3-year post-op, a pediatric neurologist confirmed the diagnosis of acquired Horner’s syndrome as a post-surgical complication, noting persistent left-sided ptosis and sensory abnormalities in the T1. Symptoms were deemed permanent. At age 13, 4-year post-op, the patient continued to experience numbness and tingling in the left upper extremity with no further treatment pursued.

### Case 3

**Fig. 6:**
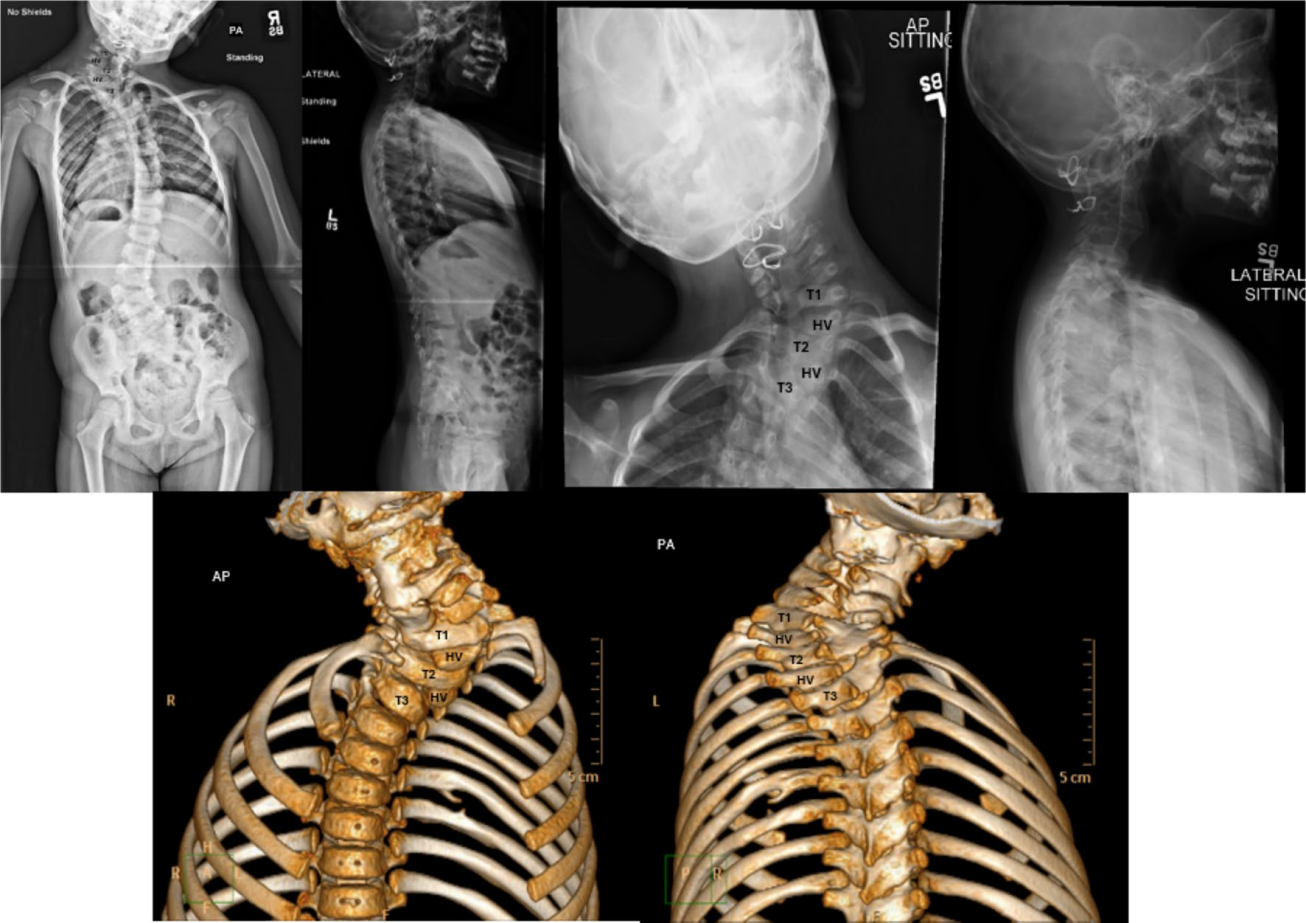
Case 3 Preoperative PA and lateral X-ray and reconstructed CT of a 4-year-old female with Klippel–Feil syndrome, C1–C2 instability, and congenital scoliosis. *HV* Hemivertebra

A 4-year-old female presented with a history of Klippel–Feil syndrome, C1–C2 instability, and congenital scoliosis with a right-sided upper thoracic hemivertebrae and left-sided lumbar hemivertebrae. At age two, she underwent posterior spine fusion of the occiput to C2 with rib autograft to address the cervical instability. Subsequently, she developed a 56-degree left-sided cervicothoracic curve with left proximal thoracic hemivertebrae between T1–T2 and T2–T3, a fixed right head tilt, and a 44-degree left lumbar curve (Fig. 6). Preoperative CT angiogram was obtained, showing an anomalous vertebral artery pattern with only one patent vertebral artery on the left.

**Fig. 7:**
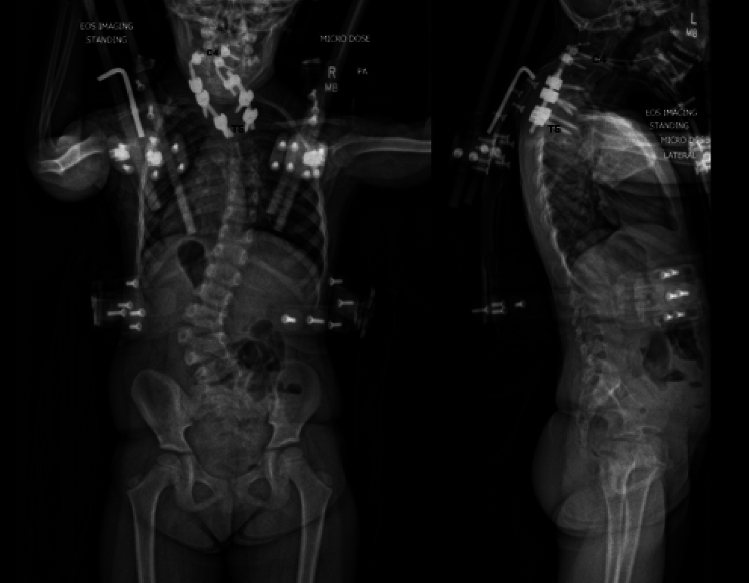
Case 3 Postoperative PA and lateral X-ray of a 4-year-old female with Klippel–Feil syndrome, C1–C2 instability, and congenital scoliosis

The patient underwent posterior spinal fusion with instrumentation from C4 to T5, including left T2 hemi vertebrectomy and halo application under general anesthesia with intra-operative neuromonitoring. During resection of the T2 left hemivertebra, the left T2 nerve root was tied off and transected to aid in the removal. Compression was applied to the resection site. No intraoperative neuromonitoring changes were noted, and a halo vest was placed postoperatively (Fig. 7).

On POD 3, the patient’s mother noted pupillary asymmetry present since surgery. Examination showed left-sided eyelid swelling with ptosis, while extraocular movements were intact, no anhidrosis was noted, and facial symmetry was preserved. Neurologic exam showed full strength at 5 out of 5, intact upper extremity sensation bilaterally, and symmetric reflexes. The patient also experienced nausea and vomiting. Neurology consultation confirmed findings aligned with Horner’s syndrome on physical exam, likely secondary to the surgical disruption of the oculosympathetic pathway from the T2 nerve root and T1 root irritation during instrumentation and curve correction. However, no pharmacologic testing or pupillometry was performed to confirm the diagnosis. The decision was made to observe, and the patient was discharged the following day.

At 2 weeks postoperatively, the patient continued to show signs of Horner’s syndrome with mild neuropathic pain in the T1 nerve distribution. At 3-month postop, ptosis resolved; however, miosis and weakness in the T1 nerve distribution persisted. MRI obtained 5 years later showed no abnormal signal intensity within the spinal cord or nerve roots. At age 12, 7 years and 7 months post-operatively, slight miosis remained, but ptosis and upper extremity weakness had resolved with no special treatments or rehabilitation performed.

## Discussion

**Fig. 8:**
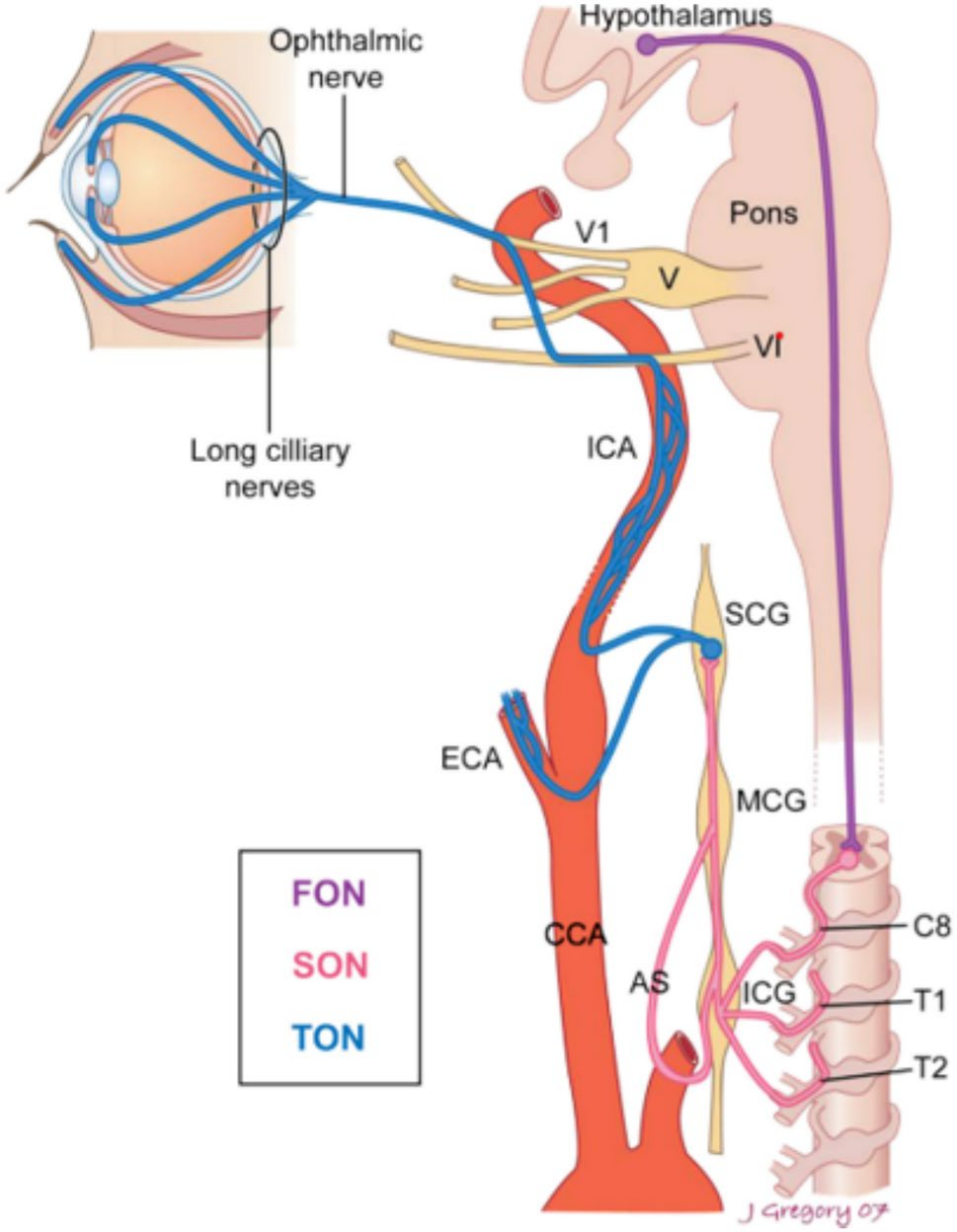
Illustrated Schematic of oculosympathic chain, obtained from Reede, Deborah L et al. Horner’s syndrome: clinical and radiographic evaluation. *AS* Ansa subclavia, *ECA* External carotid artery, *ICA* Internal carotid artery, *ICG* Inferior cervical ganglion, *MCG* Middle cervical ganglion, *SCG* Superior cervical ganglion, *FON* First-order neuron, *SON* Second-order neuron, *TON* Third-order neuron

Horner’s syndrome is a rare complication that occurs following a disruption to the oculosympathetic pathway, which extends from the hypothalamus to the eye (Fig. 8). Disruption of any part of this three-neuron track can result in the classic triad of miosis, ptosis, and anhidrosis. In the pediatric population, Horner’s syndrome may be congenital or acquired. Up to 70% of cases diagnosed within the first year of life are considered idiopathic, with birth trauma a common cause [5]. Other congenital cases are attributed to intrauterine pathologies, such as neuroblastoma. In a large cohort study, 40% of pediatric cases were congenital, 42% were acquired after a thoracic/neck/central nervous system surgery, and 15% were secondary to malignancies, such as neuroblastoma or spinal cord tumors, as well as trauma or vascular malformations [6]. Among the acquired cases, thoracotomy was the most reported surgical cause. While the incidence of Horner’s syndrome has been reported at 0.06% following anterior cervical spine surgery in all patients [2], no published studies quantify its incidence following pediatric posterior cervical or thoracic spinal surgery. A PubMed search identified five individual case reports of Horner’s syndrome postoperative complication following pediatric orthopedic spine surgery, as highlighted in Table 2. The first reported pediatric case of Horner’s syndrome following posterior spinal fusion was published by Mueller et al. in 2000, describing a 14-year-old female with idiopathic scoliosis whose diagnosis was initially delayed due to postoperative facial edema [7].

**Table 2.** Summary of systematic search on PubMed of pediatric cases of Horner’s syndrome following orthopedic spine surgery

Article #	Title	Author	Age	Sex	Comorbidities	Scoliosis classification	Surgery description	Approach	Spinal levels	Symptoms	Outcome	Duration
1	A Rare complication of Thoracic Spine Surgery: Pediatric Horner’s syndrome after posterior vertebral column resection-A case report	Grabala	5 Years	F	None	Congenital Scoliosis with severe Kyphoscoliosis	Posterior vertebral column resection with posterior pedicle screw stabilization and fusion	Posterior	C7-T9	Left-sided ptosis, miosis, and anihidrosis	Complete resolution	6 months
2	Post-operative Horner’s syndrome: A rare complication following posterior scoliosis surgery in a patient with syringomyelia	May	15 Years	M	None	Scoliosis with asymptomatic syringomyelia	Posterior pedicle screw insertion and fusion	Posterior	T4-L4	Right-sided ptosis, miosis, and anihidrosis	Complete resolution	6 months
3	Horner’s syndrome after posterior spinal fusion in a child: A case report	Mueller	14 Years	F	None	Idiopathic Scoliosis	Posterior spinal fusion	Posterior	T4-L2	Left-sided ptosis, miosis	Mild residual ptosis	–
4	Posterior-only Hemivertebra resection for congenital cervicothoracic scoliosis: correcting neck tilt and balancing the shoulders	Chen	7 Years	M	–	Scoliosis with hemivertebra	Hemivertebra resection and fusion	Posterior	C7-T5	–	Complete resolution	3 months
5	Persistent Horner’s syndrome after spinal fusion and epidural analgesia: A case report	Hered	12 Years	F	None	Idiopathic Scoliosis	Posterior spinal fusion with epidural analgesia	Posterior	T2-L2	Left-sided ptosis, miosis	Surgical repair of ptosis	6 months

Our retrospective case series describes three female patients who developed Horner’s syndrome after orthopedic spine surgery at a single pediatric orthopedic hospital, who were monitored through clinical observation by either the orthopedic team or neurology outpatient services, with an average follow-up of 5.3 years (range 2–7 years). Our first case is unique for the patient’s underlying DiGeorge syndrome, a genetic disorder involving a chromosomal microdeletion that affects embryonic pharyngeal pouch development. This leads to craniofacial anomalies, thymic and parathyroid hypoplasia, and cardiac malformations [8]. While this patient had an atrial septal defect and a patent ductus arteriosus, other common cardiac anomalies in DiGeorge syndrome include a right-sided aortic arch, duplicated aortic arch, or vascular ring [9]. These abnormalities can displace or compress components of the oculosympathetic pathway, as the second neuron passes underneath the aorta on route to the superior cervical ganglion, increasing its vulnerability during spinal surgery. This patient was noted to have both changes in both MEP and SSEPs during the right-sided lateral mass screw placement at C5–C6. MEP and SSEPs are unable to detect changes in the sympathetic pathway thus, it is unknown if these changes were predictive of development of Horner during instrumentation. SSEPs from the ulnar nerve were affected, which have contributions from C8 and T1, lower than the levels being instrumented at the time of the surgery. Motor changes were in the abductor hallucis and the abductor pollicus brevis. Of note, both SSEPs and MEPs returned to baseline before the end of surgery, with no nerve root involvement shown on the post-operative MRI.

Our second patient had congenital scoliosis with multiple vertebral anomalies. A prior attempt at thoracic posterior spinal fusion was aborted due to neuromonitoring changes to the lower extremity. Although there is no direct evidence that previous IONM changes increase the risk of complications in subsequent surgeries, such findings could indicate a predisposition for the patient. Importantly, IONM does not detect changes in the sympathetic system unless nerve root involvement occurs. Similar to Mueller’s case, diagnosis was also delayed due to masking by facial edema. Facial edema is a common post-operative complication prone positioning during surgery but has been documented to obscure Horner’s syndrome in additional case reports [4, 10]. As a result, clinicians should maintain a high index of suspicion of sympathetic pathway injury when facial edema appears abnormal or asymmetric.

Unlike Cases 1 and 2, where the injury may have been stretch or traction-related, Case 3 involved a clear iatrogenic transection of the nerve root of T2 to facilitate successful hemi-vertebra removal. At the time, neurologic deficits were anticipated, but the specific type of deficit was unclear. Yokogawa previously looked at the effect of a T2 nerve root transection, with or without T1 involvement, on upper extremity motor function. They found that transection of the T2 nerve root alone did not result in upper extremity motor dysfunction, although extensive dissection involving T1 and upper nerve roots increased the likelihood of dysfunction [11]. Chen et al. described having a patient experiencing Horner’s syndrome following a posterior hemivertebra resection between C7 and T1 for congenital cervicothoracic scoliosis. While they did not transect the C8 or T1 nerve directly, they attributed the symptoms to an indirect injury to the sympathetic chain when the hemivertebra was resected [12]. To our knowledge, there has not been a documented episode of Horner’s syndrome because of a T2 transection. However, this case shows a possible consequence of a T2 nerve root transection during a hemivertebrae removal.

This case series has limitations. Although Horner’s syndrome is primarily a clinical diagnosis, none of the three cases of Horner were confirmed with pharmacological confirmatory testing, which could have provided diagnostic confirmation and localization. In all cases, the diagnosis was based on clinical findings of ptosis and miosis, which may be challenging to detect in the immediate postoperative period due to facial or eyelid edema common from prone positioning, as previously mentioned. In addition, anhidrosis, the third symptom of the Horner’s syndrome triad, was not detected or documented in any of the three cases, which may highlight these cases as partial or incomplete syndromes. Finally, because of the retrospective nature of this series and the rarity of this complication, the exams, imaging, and neuromonitoring data were limited as they were obtained for clinical purposes and not as a standardized research assessment. These factors may introduce variability in the diagnostic certainty of each case and limit the ability to precisely determine the mechanism of injury in each case.

Together, these three cases illustrate the spectrum of etiologies underlying Horner’s syndrome following a pediatric posterior spinal fusion, from a subtle traction-related injury to overt transection. Each case emphasizes a different vulnerability of the oculosympathetic pathway during deformity correction and builds upon the limited literature regarding Horner’s syndrome as a rare but possible postoperative complication following posterior spinal fusion in children. These findings highlight the importance of awareness of this rare complication, particularly when operating near the lower cervical/upper thoracic spine. Early recognition is necessary to avoid unnecessary imaging and interventions and to counsel families on prognosis and potential outcomes.

## Data Availability

Data will not be available.

## Declarations

### Use of clinical photographs

Clinical photographs included in the study were collected under a valid Media Authorization Form previously signed by the participants, permitting their use for research and educational purposes.

## References

[1] Martin TJ, Corbett JJ (2013) The pupil. in practical neuroophthalmology, pp 261−286, McGraw-Hill Education/Medical

[2] Grabala P, Danowska-Idziok K, Helenius IJ (2023) A rare complication of thoracic spine surgery: pediatric Horner’s syndrome after posterior vertebral column resection-a case report. Children 10(1):156. 10.3390/children10010156 36670706 10.3390/children10010156 PMC9857723

[3] May IJ, Berg AJ, Dillon D (2022) Post-operative Horner’s syndrome: a rare complication following posterior scoliosis surgery in a patient with syringomyelia. Cureus 14(5):e25242. 10.7759/cureus.25242 35755498 10.7759/cureus.25242 PMC9217680

[4] George ND, Gonzalez G, Hoyt CS (1998) Does Horner’s syndrome in infancy require investigation? Br J Ophthalmol 82(1):51–54. 10.1136/bjo.82.1.51 9536881 10.1136/bjo.82.1.51 PMC1722330

[5] Jeffery AR, Ellis FJ, Repka MX, Buncic JR (1998) Pediatric Horner syndrome. J Am Assoc Pediatr Ophthalmol Strabismus 2:159–167. 10.1016/s1091-8531(98)90008-8 10.1016/s1091-8531(98)90008-8 10532753

[6] Smith SJ, Diehl N, Leavitt JA, Mohney BG (2010) Incidence of pediatric Horner syndrome and the risk of neuroblastoma: a population-based study. Arch Ophthalmol 128(3):324–329. 10.1001/archophthalmol.2010.6 20212203 10.1001/archophthalmol.2010.6 PMC3743544

[7] Mueller KL, Loder RT, Eggenberger ER, Farley FA (2000) Horner’s syndrome after posterior spinal fusion in a child: a case report. Spine 25(21):2836–2837 11064532 10.1097/00007632-200011010-00019

[8] Altshuler E, Saidi A, Budd J (2022) DiGeorge syndrome: consider the diagnosis. BMJ Case Rep 15(2):e245164. 10.1136/bcr-2021-245164 35110278 10.1136/bcr-2021-245164 PMC8811567

[9] McDonald-McGinn DM, Sullivan KE (2011) Chromosome 22q11.2 deletion syndrome (DiGeorge syndrome/Velocardiofacial syndrome). Medicine 90(1):1–18. 10.1097/MD.0b013e3182060469 21200182 10.1097/MD.0b013e3182060469

[10] Ongaigui C, Fiorda-Diaz J, Dada O, Mavarez-Martinez A, Echeverria-Villalobos M, Bergese SD (2020) Intraoperative fluid management in patients undergoing spine surgery: a narrative review. Front Surg 7:45. 10.3389/fsurg.2020.00045 32850944 10.3389/fsurg.2020.00045 PMC7403195

[11] Yokogawa N, Murakami H, Demura S, Kato S, Yoshioka K, Hayashi H, Ishii T, Fujii M, Igarashi T, Tsuchiya H (2014) Motor function of the upper-extremity after transection of the second thoracic nerve root during total en bloc spondylectomy. PLoS ONE 9(10):e109838. 10.1371/journal.pone.0109838 25333299 10.1371/journal.pone.0109838 PMC4198131

[12] Chen Z, Qiu Y, Zhu Z, Li S, Chen X, Xu L, Sun X (2018) Posterior-only hemivertebra resection for congenital cervicothoracic scoliosis: correcting neck tilt and balancing the shoulders. Spine 43(6):394–401. 10.1097/BRS.0000000000002325 28700454 10.1097/BRS.0000000000002325

[13] Gagnier JJ, Kienle G, Altman DG, Moher D, Sox H, Riley D (2013) The CARE guidelines: consensus-based clinical case reporting guideline development. BMJ Case Rep. 10.1136/bcr-2013-201554 24228906 10.1186/1752-1947-7-223 PMC3844611

